# Trapezoid-kinoform zone plate lens – a solution for efficient focusing in hard X-ray optics

**DOI:** 10.1107/S1600577522000893

**Published:** 2022-02-15

**Authors:** Xujie Tong, Yifang Chen, Zijian Xu, Yijie Li, Zhenjiang Xing, Chengyang Mu, Jun Zhao, Xiangjun Zhen, Chengwen Mao, Renzhong Tai

**Affiliations:** a Nanolithography and Application Research Group, School of Information Science and Technology, Fudan University, Shanghai 200433, People’s Republic of China; b Shanghai Synchrotron Radiation Facility, Shanghai Advanced Research Institute, Chinese Academy of Sciences, Shanghai 201210, People’s Republic of China

**Keywords:** kinoform zone plate lens, 3D greyscale electron beam lithography, focusing efficiency, X-ray microscopy, beam propagation method with Hankel transform

## Abstract

X-ray microscopes are powerful tools in the nano-inspection of materials owing to their ultra-high resolution at the molecular level. However, the focusing efficiency of binary zone plate lenses as key components in such probes is merely 5% in practice, hindering their application in advanced scientific research. Although kinoform zone plate lenses are in principle supposed to possess high efficiency beyond binary ones, little progress has been reported so far due to the shortage of both a theoretical calculation approach and greyscale lithography for generating fine three-dimensional (3D) kinoform zones of the lenses. This paper reports our theoretical work for a modified beam propagation method to compute the focusing performance and state-of-the-art 3D greyscale electron beam lithography for kinoform zone plate lenses. Three different zone shapes – binary, kinoform and top-flat kinoform (nicknamed the trapezoid-kinoform) – were compared both theoretically and experimentally. Theoretical calculations suggest, for the first time, that the trapezoid-kinoform zone plate gives rise to the highest focusing efficiency among the three lenses, which was proved by optical characterization of the fabricated lens with hard X-rays. As high as 40% of the focusing efficiency by Au trapezoid-kinoform lenses with resolution of 250 nm at 8 keV has been achieved, which is two times higher than that of binary zone plate lenses. The origin of the enhanced efficiency in the trapezoid-kinoform zone plate lens was explained by the joint contributions from both the refraction through the kinoform slope and the diffraction through the top flat part of the trapezoid-kinoform zone plate. Such a breakthrough in focusing efficiency sheds light on the further development of X-ray lenses with both high resolution and high efficiency.

## Abstract 2

A new theoretical approach for computing the focusing performance of X-ray optics with circularly symmetry shape was developed. Greyscale electron beam lithography was applied to generate 3D kinoform zones in Au for hard X-rays for the first time. The novel trapezoid kinoform zone plate lens demonstrates significantly high diffraction efficiency well beyond that of conventional X-ray optics.

## 1. Introduction

Fresnel zone plate (FZP) lenses as diffractive X-ray optics are key components in X-ray microscopes such as the full-field transmission X-ray microscope (Chu *et al.*, 2008 ▸) and the scanning transmission X-ray microscope (Kirz, 1974 ▸; Spector *et al.*, 1997 ▸) for nanoscale focusing and imaging (Sakdinawat & Attwood, 2010 ▸; Chen *et al.*, 2021 ▸; Oktem *et al.*, 2018 ▸; Fan *et al.*, 2018 ▸). Despite frequent reports of resolutions as high as 10 nm (Mohacsi *et al.*, 2017 ▸; Rösner *et al.*, 2018 ▸), binary FZP lenses still suffer from low diffraction efficiency, typically 1–5% (Sanli *et al.*, 2018 ▸; Moldovan *et al.*, 2018 ▸; Mohacsi *et al.*, 2017 ▸), even with impressively high aspect ratios (zone-width/zone-height) (Vila-Comamala *et al.*, 2009 ▸; Uhlén *et al.*, 2011 ▸; Mohacsi *et al.*, 2017 ▸; Rösner *et al.*, 2018 ▸).

In the past decades, zone plates with parabolic thickness profiles in kinoform zone plate (KZP) lenses have attracted considerable attention, inspired by the expectation of the focusing efficiency of up to 100% (Di Fabrizio *et al.*, 1994 ▸) in perfectly transparent materials. Unfortunately, limited progress has been witnessed owing to the shortage of not only theoretical works to establish the relation between the focusing/imaging efficiency and the zone structure but also a fine greyscale lithography technique to generate the desired 3D zone shape of the lenses.

In theoretical studies, an angular spectrum method for calculating the focusing/imaging performance of multi-stage zone plate lenses was reported by Vila-Comamala and co-workers (Di Fabrizio & Gentili, 1999 ▸; Yun *et al.*, 1999 ▸; Di Fabrizio *et al.*, 1994 ▸; Vila-Comamala *et al.*, 2013 ▸), which is, however, not capable of dealing with a kinoform profile with a continuous change of zone height. The beam propagation method (BPM) was applied for analyzing the focusing/imaging of a one-dimensional kinoform lens only (Yan, 2010 ▸). In experimental studies, non-rectangular zone plates, such as the staircase zone plate by multistep e-beam lithography (Takeuchi *et al.*, 2012 ▸), parabolically shaped zone plates by focused ion beam (Keskinbora *et al.*, 2015 ▸) and femtosecond two-photon 3D nanoprinting (Sanli *et al.*, 2018 ▸) have been attempted. However, limited by the lithography capability, these techniques suffer from either modest efficiency or poor resolution. Using greyscale-focused Xe ion beam lithography, a zone plate lens with nonvertical and short zones was fabricated, that shows an even lower focusing efficiency than binary ones in the 5–7 keV range (Gorelick *et al.*, 2019 ▸), leading to controversial conclusions for the prospect of KZP lenses. It is therefore urgently necessary to conduct systematical research on both a theoretical approach and reliable 3D greyscale lithography for a new generation of zone plate lens with high resolution as well as efficient focusing efficiency for X-ray microscopy as a whole.

This paper reports our recent progress on developing a new fashion of KZP lens, nicknamed the trapezoid-kinoform zone plate (TKZP) lens, with a flat top for each zone, toward the solution for efficient efficiency. By modifying the BPM, a theoretical approach for computing the focusing performance of a rotationally symmetric zone plate lens with arbitrary shape was established. 3D greyscale electron beam lithography (GS-EBL) was successfully applied in generating theoretically designed TKZPs with necessary heights. Optical characterization of the fabricated TKZP lenses using hard X-rays demonstrates a focusing efficiency twice as high as that of a binary zone plate lens, in close agreement with theoretically calculated figures. The enhancement of the focusing efficiency in the TKZP lens was theoretically explained by joint contributions from refracted light through the kinoform slope and diffracted light through the flat top of the zones. The success in this work opens up a promising avenue toward X-ray focusing/imaging with high resolution and high efficiency.

## 2. Constructing the TKZP lenses

**Figure 1:**
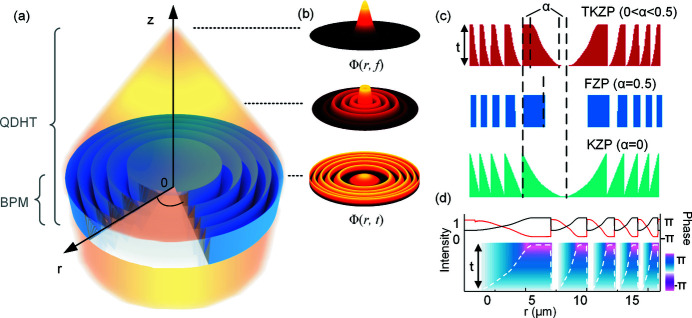
(*a*) Schematic illustration of the computation approach developed in this work – the traditional beam propagation method combined with the quasi-discrete Hankel transform (BPM-QDHT) in three dimensions. (*b*) The optical wavefield was calculated by BPM-QDHT to obtain Φ(*r*, *t*) at the exit of the KZP lens. The wavefield Φ(*r*, *f*) at the focal plane is calculated using diffraction theory and QDHT in free space. (*c*) The topography of the binary Fresnel zone plate (FZP), kinoform zone plate (KZP) and trapezoid-kinoform zone plate (TKZP). (*d*) Partial 2D phase map of the wavefields inside the TKZP with diameter of 100 µm, outermost zone width of 500 nm and thickness of 1.8 µm, and wavefield intensity (red line) and phase (black line) at the exit of the lens. The white dashed line represents the zone profile.

For an X-ray lens with 3D profile as illustrated in Figs. 1 ▸(*a*) and 1(*b*) in this work, the optical wavefield transmission through the lens was calculated by the BPM based on the quasi-discrete Hankel transform (QDHT). The initial wavefield inside the KZP lens should be first established before working out that in the far-field. Supposing the KZP lens was illuminated by a plane wave Φ(*r*, 0) with unit amplitude, for light propagating in the *z*-direction inside the lens over a small distance *h*, the electric field at *z* + *h* can be calculated using the BPM (Yan, 2010 ▸) as

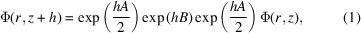




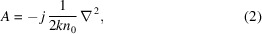




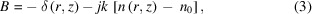

where the refractive index of the lens material is defined as *n* = 1 − δ + *j*β, in which δ and β are the dispersive and the absorptive properties of the material, respectively (see Section S1 of the supporting information for details). Since the two-dimensional X-ray lenses in this work have rotational symmetry, we propose to replace the fast Fourier transform in the BPM by the QDHT in the calculation of the near-field wavefield. A QDHT (Guizar-Sicairos & Gutiérrez-Vega, 2004 ▸; Yu *et al.*, 1998 ▸) routine helps to simplify the model of the circular symmetric kinoform lens and to increase the calculation speed. The Fourier spectrum Ψ(*f*_*x*_, *f*_*y*_, *z*) of an optical wavefield Φ(*x*, *y*, *z*) at the plane (*z*) can be expressed as a simplified one-dimensional zero-order Hankel transform (Goodman, 1996 ▸),

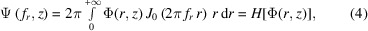

where *J*_0_ is the zero-order Bessel function of the first kind. Taking advantage of the rotational symmetry, the BPM combined with the quasi-discrete Hankel transform (BPM-QDHT) can be extended to any 3D shape of the zones, provided that the rotational symmetry is preserved, for fast and effective calculations of both the near- and the far-field wavefields of an X-ray lens.

The established computation approach is applied in systematic comparisons of three different topographies of the zone plate lens – the kinoform (KZP), the binary (FZP) and the kinoform with a flat top, termed the trapezoid-kinoform (TKZP) – as schematically illustrated in Fig. 1 ▸(*c*). In this work, the lenses are designed to provide a focal length of 161 mm at 8 keV with a diameter of 100 µm and an outermost zone width of 500 nm. The thickness *t* of the KZP and TKZP is 1.8 µm and that of the FZP is 1.5 µm. Due to the finite spatial resolution of the lithography, the sharp edge of the KZP is difficult to achieve so that fabricated KZP lenses always turn out to be TKZP lenses. Therefore, such TKZPs lenses are mainly targeted in this research.

The wavefields inside the three lenses were calculated for the first time using the BPM-QDHT approach developed in this work. To mathematically distinguish the difference between the three different lenses, a structural factor α, defined as the ratio of the flat region-width against the kinoform region-width in each zone, is introduced, for which 0 ≤ α ≤ 0.5. Obviously, as illustrated in Fig. 1 ▸(*c*), α = 0 denotes the binary FZP, α = 0.5 the KZP, and 0 < α < 0.5 the TKZP. Fig. 1 ▸(*d*) presents parts of the calculated wavefield intensity (red line) and the phase (black line) for a TKZP lens of diameter 100 µm, outermost zone width of 500 nm and thickness of 1.8 µm, which is depicted by the white dashed line, showing a diffraction–refraction mixed focusing mechanism. The phase distribution in regions with kinoform profiles behaves more like a refractive lens, whilst that on the flat top of the TKZP is similar to that of the FZP (see Section S1 of the supporting information), which interferes constructively at the focus. Furthermore, the unwanted diffraction caused by sharp edges in the FZP should no longer exist in a TKZP lens (Yan, 2010 ▸). Therefore, all the zones including those in the central area of the TKZP contribute to focusing incoming light, in contrast to those in the FZP in which a great number of central zones fail to contribute to light focusing.

**Figure 2:**
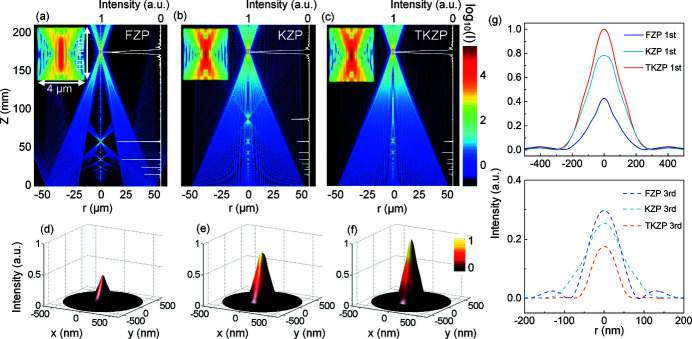
The focusing properties through the binary FZP (*a*, *d*), KZP (*b*, *e*) and TKZP (*c*, *f*) are numerically calculated by our BPM-QDHT approach for an X-ray energy of 8 keV. The lenses have a focal length of 161 mm at 8 keV with diameter of 100 µm and outermost zone width of 500 nm. The thickness *t* of both the KZP and TKZP is 1.8 µm, and is 1.5 µm for the FZP to obtain the best efficiency. (*a*–*c*) The 2D intensity (on logarithm scale) of the propagated wavefield created by three different types of zone plate lenses. In the insets, the 1D curves (white lines) are the normalized intensity distribution along the axis. (*d*–*f*) The normalized 3D surface profile of the first-order focus intensity of three different types of zone plate lenses. (*g*) The normalized intensities of the first-order focal spots and the third-order focal spots of the three lenses. (All the design parameters are given in Table S1 of the supporting information.)

The propagation in free space to the focal plane [from Φ(*r*, *t*) to Φ(*r*, *f*)] can be obtained by multiplying the angular spectrum of the initial wavefield, as described by Vila-Comamala *et al.* (2013 ▸). Fig. 2 ▸ presents the calculated characteristics of the focusing performance in free space for lenses with three different ring structures as mentioned above at 8 keV – the binary FZP (*a*), the KZP (*b*) and the TKZP (*c*). First, well defined first-order focusing points [Fig. 2 ▸(*a*)–2(*c*)] of the three lenses indicate that a beamstop in a KZP and/or TKZP lens is not necessary in contrast to the binary FZP, in which a beamstop in the center is a must to block the directly transmitted light. Second, the TKZP lens exhibits the highest efficiency among the three [Figs. 2 ▸(*d*)–2(*f*)], which is 25% higher than that of the KZP and twice that of the FZP, as shown in Fig. 2 ▸(*g*). As mentioned above, the flat top of the trapezoid-kinoform zone [Fig. 1 ▸(*c*)] helps to reduce the edge-induced interference fringes that may degrade the efficiency (Yan, 2010 ▸). Third, a significant difference in energy distribution in foci orders between the three different kinds of lenses is observed. Unlike the FZPs, in which the higher focusing orders take considerable amounts of energy, the TKZP holds more than 30% intensity in the first focusing order, leaving limited energy to higher orders. For example, the second focusing order takes less than 1% of the focusing efficiency only, as presented by the insets in Fig. 2 ▸(*g*). Therefore, the TKZP developed in this work should be a promising candidate for the next generation of X-ray lenses, exhibiting the highest focusing/imaging efficiency.

**Figure 3:**
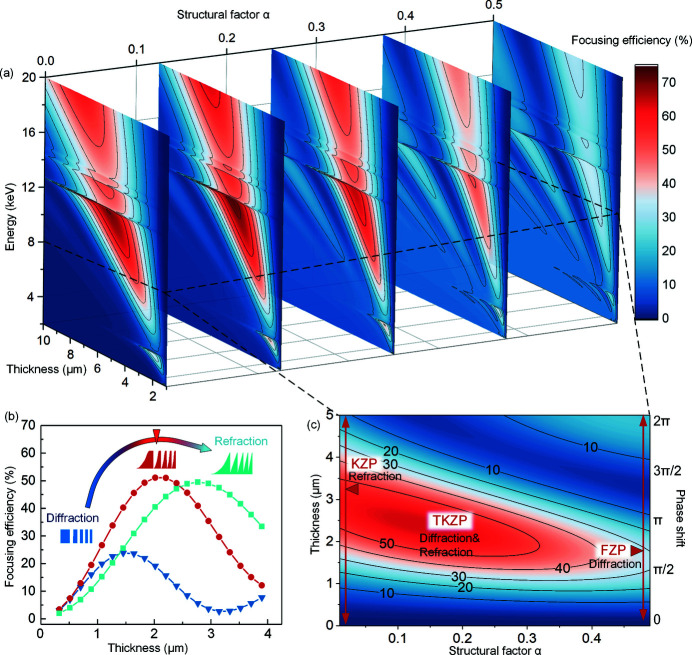
Calculated focusing efficiency (FE) of three different types of zone plate lenses. (*a*) The theoretical focusing efficiency of the TKZP as a function of the structural factor α, photon energy and lens thickness, based on the thin grating approximation. The map shares the same color bar with (*c*). (*b*) The focusing efficiencies of the FZP, KZP and TKZP (α = 0.2) calculated by the QDHT-BPM approach at 8 keV. (*c*) Focusing efficiency map in the plane of the structural factor α and the lens thickness *t* at 8 keV, obtained by QDHT-BPM.

The theoretical calculation of the focusing efficiency of zone plate lenses can be performed by summing the contributions from all of the zones. The thin grating approximation described by Kirz (1974 ▸) was applied. The sum of amplitudes at the first order *A*_1_ for the TKZP is given by

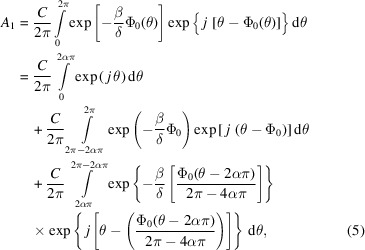

where *C* is the incident amplitude, Φ_0_ = 2π*t*δ/λ is the phase shift in the material with thickness *t*, and θ is the optical path length difference to the focal spot over one ring period. Then, the focusing efficiency (FE) should be given by FE = *A*_1_^2^/*C* ^2^ × 100%. The theoretical FEs of the TKZP as a function of the structural factor α, photon energy and lens thickness are presented in Fig. 3 ▸(*a*). The theoretical FE maps with different α show that TKZPs (0 < α < 0.5) can achieve a higher peak focusing efficiency than the KZP or FZP does with smaller thickness. For comparison, the FEs of the three lenses are also calculated by the BPM-QDHT method as shown in Fig. 3 ▸(*b*), where FE is defined as the integral ratio of the light intensity on the focal plane diffracted from the lens over a distance *f* and the incident *I*_0_ in the whole range of the lens with the radius *R*, 

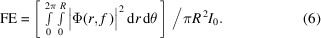

First of all, efficiencies as high as 45–50% for the two lenses TKZP and KZP in Au can be reached. However, that of the FZP is merely half of that of the kinoform lenses as expected. The optimized thickness of the FZP is 1.5 µm under 8 keV of the photon energy with an efficiency of 23%. Second, the thickness *t* of the TKZP only needs to be 1.8 µm for a focusing efficiency of 46%, which is 33% thinner than that of the KZP lens (*t* = 2.7 µm), significantly easing the fabrication processing of the TKZP lens. Third, when the lens thickness *t* satisfies integers of π phase shift (*m*λ/2δ) or even smaller, the diffractive focus ability of the FZPs is strong while the kinoform profiles (KZP) are still weak in the modulating wavefront, which explains the poor performance of thin KZPs (Gorelick *et al.*, 2019 ▸). Finally, when the thickness is doubled to satisfy a 2π phase shift (*m*λ/δ), the diffraction focusing mechanism through the top flat part of the TKZP is suppressed and the refractive one by the kinoform part dominates with high focusing efficiency. Therefore, the TKZP lens certainly exhibits superior performance in focusing efficiency over the other two types of lenses. A more comprehensive and quantitative comparison can be seen in Fig. 3 ▸(*c*), which presents the theoretical efficiency (FE) map in hard X-rays for 8 keV in the α–*t* plane. In the map, the top border, α = 0.5, corresponds to the FZPs, and the bottom one, α = 0, to the KZPs. The overall area inside the borders is defined by 0 < α < 0.5 for the TKZPs, which is closer to the fabricated ones than the KZPs. It is clear that from the FZPs to the KZPs the focusing efficiency undergoes a significant increment with the peak in the large thickness range for TKZPs, indicating that the TKZP lens proposed in this work possesses great advantage over the other two in focusing efficiency.

## 3. Fabrication and optical characterizations of TKZPs

**Figure 4:**
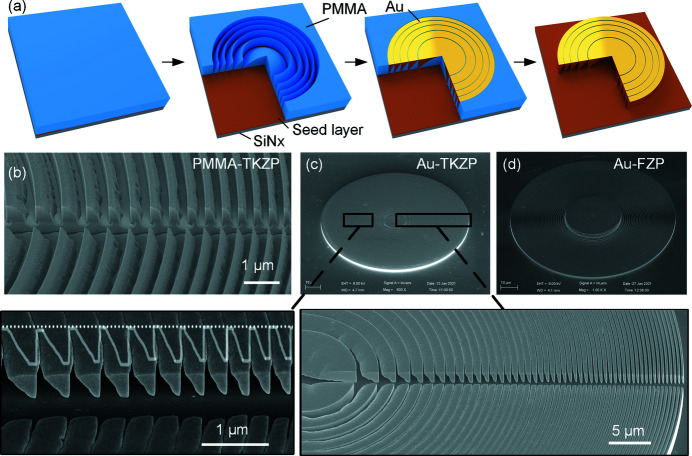
Overview of the fabrication procedures. (*a*) Schematic illustration of GS-EBL for the Au-TKZP lens with diameter of 100 µm and outermost zone width of 250 nm. (*b*) SEM micrographs at a tilt of 45° for the replicated TKZP profiles in PMMA as a template for Au electroplating. (*c*) The fabricated TKZP lens in Au. (*d*) The fabricated FZP lens with a beamstop after gold electroplating. A 2 µm-wide trench crossing the center of the plate was deliberately arranged in testing samples to inspect the trapezoid kinofom profiles. White lines are the digitized curves (Au-TKZP+) of the actual zone profiles of Au-TKZP.

In experiments, 3D kinoform-shaped zone plate lenses were replicated by GS-EBL for the first time, using a state-of-the-art e-beam writer, JBX6300 FS, supplied by Jeol Ltd. Fig. 4 ▸ (*a*) illustrates the GS-EBL process flow. To fabricate Au-TKZP lenses with a diameter of 100 µm and outermost zone width of 250 nm for hard X-rays (see parameters in Table S1 of the supporting information), a 2 µm-thick PMMA was first spin-coated on a 100 nm-thick Si_3_N_4_ membrane with a seeding layer of 5 nm Cr/10 nm Au. To achieve the parabolic shape for the zones by GS-EBL, exposure doses were assigned to the TKZP pattern according to the calculated exposure levels for each ridge (details can be seen in the supporting information). Greyscale exposure was carried out using the JBX-6300 FS at 100 kV with an e-beam of 7 nm in diameter and 500 pA as the beam current. Mild developing solution (IPA:H_2_O = 7:3) was applied in the development to form the desired profile with a smooth surface (Chen, 2015 ▸; Tong *et al.*, 2020 ▸). The replicated TKZP shapes in PMMA with outermost zone-width of 500 nm are presented in Fig. 4 ▸(*b*). Clear parabolic-like profiles can be observed. Au-TKZP lenses were then formed by gold electroplating in a K_3_Au(SO_3_)_2_ electrolyte (10 g L^−1^ concentration, 50°C), using the patterned resist as the template. A constant current of 3 µA supplied by a current source (Keithley Ltd 2400) was applied in the plating for 15 min, followed by a lift-off process in acetone. The upside-down trapezoid-kinoform structures are shown in Fig. 4 ▸(*c*) from a 2 µm-wide trench, which was opened through the center of the plate during the process for profile inspection purposes only. The fabricated Au-TKZP lens is arranged upside-down to prevent the patterned PMMA template from collapsing before Au electroplating. An Au-FZP with beamstop was also prepared for comparison. Almost all of the zones in the fabricated lenses have trapezoid-kinoform shape thanks to the optimized dose allocation and proximity effect in GS-EBL, which is actually beneficial in this case for continuous height change as desired. However, it can be seen in the magnified image in Fig. 4 ▸(*c*) that the proximity effect in the outer area of the lens with small periods would apply a negative impact on the replicated profiles by deforming the trapezoid ridges into tips with vertical sidewall. The actual zone profile of the fabricated TKZPs, which is outlined by the white line in Fig. 4 ▸(*c*), is named TKZP+ for further calculation. The sidewalls of the Au-TKZP+ in the outer area are steeper than that of the designed one and may cause unwanted diffraction, which will be discussed later.

Focusing efficiency calculations of the fabricated TKZP lenses were carried out at Shanghai Synchrotron Radiation Facility, using in-house-developed scanning transmission X-ray microscopes with hard X-rays at beamline BL15U1. Detailed descriptions of the measurement setup can be found in the supporting information. Standard testing samples, *i.e.* Siemens stars, were prepared in-house with a resolution of 100 nm and height of 2.5 µm, suitable for hard X-rays test.

**Figure 5:**
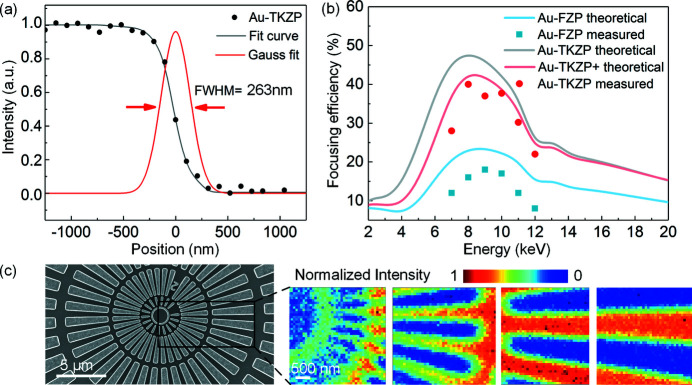
Characterizations of the focusing performance of the fabricated Au-TKZP. (*a*) The focusing beam profiles of the Au-TKZP lens, measured by the knife-edge scanning method with dwell time of 100 ms and step size of 100 nm. The dotted line is the raw data, the solid line in black is the least-squares fitting curve and the red line is the first derivative of the fitted scanning curve, giving rise to a the full width at half-maximum of 263 nm for the Au-TKZP lens. (*b*) The measured focusing efficiencies of Au-TKZP and Au FZP. For comparison, their corresponding theoretical figures are also presented. The efficiency labeled TKZP+ was calculated from the actual zone profiles. (*c*) SEM image (left) of the in-house-made Siemens stars with minimal resolution of 100 nm and X-ray image (right) of the same target at 8 keV with a dwelling time of 100 ms and step size of 150 nm.

Fig. 5 ▸(*a*) presents the measurement results of the focusing spot dimensions using the knife-edge method by scanning the focused spot on the line edge of the Siemen star. The measured full width at half-maximum is 263 nm, very close to the designed value. The focusing efficiency was characterized by measuring the photocurrents from the photodetector. The efficiency was determined by

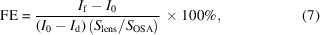

where *I*_0_ is a blank window of the chip for the incident flux; *I*_f_ is the current after focusing with the lens and OSA in the optical path; *I*_d_ is the dark current with the beam blocked. *S*_OSA_ and *S*_lens_ are the area of the aperture spot and the objective lens, respectively.

The measured efficiencies from the fabricated Au-TKZP and the Au-FZP together with the theoretically calculated results using our BPM-QDHT algorithm are presented in Fig. 5 ▸(*b*). Based on the actual zone profiles of the fabricated TKZPs, which were obtained from the scanning electron microscope (SEM) images in Fig. 4 ▸(*c*), the calculated efficiency (Au-TKZP+) is also added to Fig. 5 ▸(*b*) for comparison. The measurement using hard X-rays [Fig. 5 ▸(*b*)] shows the FE of the Au-TKZP peaks at 40% at 8 keV, which agrees well with the calculated FE of Au-TKZP+ but is about 83% of the theoretical one of Au-TKZP, caused by the structural deviations between the TKZP+ and the designed TKZP. The focusing efficiency of the Au-FZP lens with outermost zone width of 250 nm and height of 1.5 µm is 18% at the same photon energy, indicating that the focusing efficiency is increased by a factor of two from binary to trapezoid-kinoform zone profile. Fig. 5 ▸(*c*) presents images of the testing samples (Siemens star) through the fabricated Au-TKZP lens by an in-house-developed scanning transmission X-ray microscope at 8 keV. High-contrast images with a minimal feature size of 270 nm can be seen, which is in agreement with the designed resolution within the measurement accuracy.

The experimental results presented in both Figs. 4 ▸ and 5 ▸ deliver a very important message for the practically feasible solution of high-efficiency focusing by zone plate lenses with hard X-rays. In principle, to maximize the focusing efficiency by a zone plate, all zones including the central ones should be kinoform shaped to diffract and/or refract the incoming light to the focal plane. However, the sharp edge of the kinoform zone diverts partial light to unwanted high orders of foci, resulting in a loss of efficiency. To eliminate such an energy loss, a flat plateau on the kinoform zone top is introduced in this work and its width, characterized by a structural factor α (plateau-width/zone-width), is theoretically optimized by our BPM approach. As shown in Fig. 3 ▸, the ideal structural factor for the peak efficiency should be α = 0.1. State-of-the-art 3D GS-EBL enables us to realize the designed TKZP lenses in Au, and their optical characterization with hard X-rays has demonstrated a breakthrough in the focusing efficiency well beyond the conventional ones.

In practice, it should be feasible to achieve a minimum line-width around 10 nm by 3D GS-EBL. Considering a structural factor of α = 0.1 for peak efficiency, the minimum zone width achievable in a TKZP should be 100 nm, anticipating that the highest resolution of the TKZP lens should be 50 nm with peak efficiency. Zone plate lenses with higher resolution than 50 nm with high efficiency demands more sophisticated nanofabrication techniques. Besides, both the established computing approach of the focusing property and 3D GS-EBL should be extendable to the development of TKZPs with enhanced focusing efficiency with soft X-rays.

## 4. Conclusion and outlook

In this work, a breakthrough for efficient focusing in hard X-ray optics has been achieved. A new zone plate lens with trapezoid-kinoform shape of the rings was developed, which has been proved both theoretically and experimentally to possess the highest focusing efficiency, comparing with the conventional binary as well as kinoform zone plate lens. For the success of this work, a new theoretical approach for computing the focusing/imaging performance of zone plate lenses of arbitrary shape with circularly symmetry was first developed, which was then applied in the optimization of lens shapes for high focusing efficiency. GS-EBL was successfully utilized to generate 3D kinoform zones at the nanoscale for the fabrication of TKZP lenses in Au for hard X-rays. A focusing efficiency of 40% has been achieved for hard X-rays with the Au-TKZP lens with 263 nm resolution. The origin of the efficiency enhancement in the Au-TKZP lens is understood as joint contributions from both the kinoform slope by refraction and from the top flat part by diffraction of all the zones including central ones, instead of being optically blocked by a beamstop as in binary zone plate lenses. The theoretical approach and the fabrication process based on 3D GS-EBL should also be applicable for soft X-ray optics, which is still under way in our work. Therefore, this work has laid a solid foundation for the development of a new generation of zone plate lenses with high resolution and high efficiency in X-ray optics as a whole.

## Supplementary Material

Supporting information: Sections S1 to S2; Figures S1 to S3; Table S1. DOI: 10.1107/S1600577522000893/ve5155sup1.pdf
