# Supplementary material for: Trapezoid-kinoform zone plate lens – a solution for efficient focusing in hard X-ray optics
Source: J Synchrotron Radiat. 2022 Feb 15;29(Pt 2):386–92. doi: 10.1107/S1600577522000893 (PMC8900836; doi:10.1107/S1600577522000893)
Supplement: Supplementary file 1 [file s-29-00386-sup1.pdf]

JOURNAL OF SYNCHROTRON RADIATION

Volume 29 (2022)

Supporting information for article:

Trapezoid-Kinoform zone plate lens, the solution for efficient focusing in hard X-ray optics

Xujie Tong, Yifang Chen, Zijian Xu, Yijie Li, Zhenjiang Xing, Chengyang Mu, Jun Zhao, Xiangjun Zhen, Chengwen Mao and Renzhong Tai

S1. Beam propagation method based on quasi-discrete Hankel transforms

Beam propagation method (BPM) based on fast Fourier transform (FFT) is a common technique to investigate one-dimensional X-ray optics with high accuracy and fast calculation speed. However, it is hard to construct models of two-dimensional X-ray lenses such as Fresnel zone plates and circular Kinoform lenses. Here, taking the specialty of circular symmetry of X-ray lenses, we propose to replace the FFT in the BPM by the quasi-discrete Hankel transform (QDHT). Hankel transform can simplify the calculation procedure by transforming the problem from three-dimensional space to two-dimensional in a cylindrical coordinate system, greatly improving the calculation efficiency, while maintaining the advantages of high degree of freedom and high accuracy of BPM. BPM based on quasi-discrete HT can be efficiently applied to calculate circularly symmetric wavefield in both near-field and far-field.

The initial wavefield inside the KZP lens should be first established before working out the one in far-field. Assuming the Kinoform zone plate lens was illuminated by a plane wave $\Phi(r, 0)$ with unit amplitude. For a light propagation at z-direction inside the lens over a small distance h, the electric field at $z+h$ can be calculated using BPM as

$$\Phi(r, z+h) = \exp\left(\frac{h\mathbf{A}}{2}\right)\exp(h\mathbf{B})\exp\left(\frac{h\mathbf{A}}{2}\right)\Phi(r, z) \tag{S1}$$

where **A** and **B** are respectively:

$$\mathbf{A} = -j\frac{1}{2kn_0}\nabla^2 \tag{S2}$$

$$\mathbf{B} = -\delta(r, z) - jk\left[n(r, z) - n_0\right] \tag{S3}$$

This equation says that the electric field $\Phi(r, z)$ is regarded as propagated first in the free space (operator **A**) over a distance of $h/2$,

$$\begin{aligned}\Phi\left(r, z+\frac{h}{2}\right) &= \exp\left(\frac{h\mathbf{A}}{2}\right)\Phi(r, z) \\ &= \boldsymbol{H}^{-1}\left[\exp\left(-\frac{j\Delta\beta h}{2}\right)\boldsymbol{H}\left\{\Phi(r, z)\right\}\right]\end{aligned} \tag{S4}$$

where $\Delta\beta = \beta - kn_0 = \left[k^2 n_0^2 - (2\pi f_r)^2\right]^{1/2} - kn_0$, $\boldsymbol{H}$ is Hankel transform. Then the loss and the phase retardation of the entire length h is taken into account at the center of propagation (operator **B**).

$$\Phi^*\left(r, z+\frac{h}{2}\right) = \exp(h\mathbf{B})\Phi\left(r, z+\frac{h}{2}\right) = \exp\left\{\frac{h}{2}\left[\mathbf{B}(z)+\mathbf{B}(z+h)\right]\right\}\Phi\left(r, z+\frac{h}{2}\right) \tag{S5}$$

This electric field is again propagated in the latter free space (operator **A**) at the distance $h/2$ to obtain $\Phi(r, z+h)$.

$$\Phi(r, z+h) = \exp\left(\frac{h\mathbf{A}}{2}\right)\Phi^*\left(r, z+\frac{h}{2}\right) = \boldsymbol{H}^{-1}\left[\exp\left(-\frac{j\Delta\beta h}{2}\right)\boldsymbol{H}\left\{\Phi^*\left(r, z+\frac{h}{2}\right)\right\}\right] \tag{S6}$$

X-ray propagation inside the KZP lens can be quantitatively figured out by repeating this computation process many times for all slices.

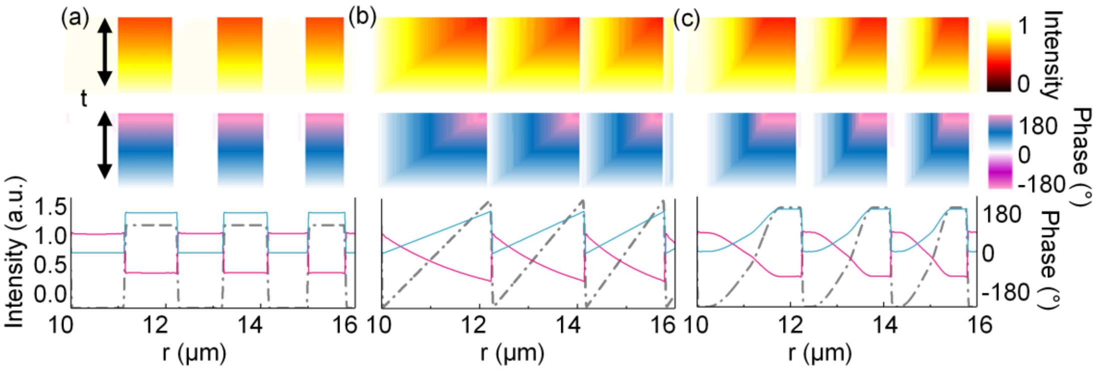

Figure S1 The wave propagation through binary Fresnel zone plate (a), Kinoform zone plate (b) and trapezoid Kinoform zone plate (c) are numerically calculated by BPM-QDHT approach for the X-ray energy of 8 keV, respectively. The structure parameters are shown in Table S1. (a-c): The calculation results are: 2D intensity (top) and 2D phase distribution (middle) of the wavefields inside the lens, the 1D wavefield intensity (red line) and the phase (blue line) at the exit of the lens (bottom). The gray dash-dotted line describes the zone profile.

S2. The details in the fabrication and optical tests of Kinoform zone plate lenses

To achieve the parabolic shape for the zones by GS-EBL, exposure doses were assigned to the KZP lens pattern according to the calculated exposure levels for each unit ring, as schematically illustrated in Fig. S3(a), Monte Carlo simulation using BEAMER/TRACER software, supplied by GenIsys Ltd, was carried out to work out the spatial distributions of the exposure dose. Proximity effect correction (PEC) by TRACER was also included throughout the simulation sequence. The optimized dose distributions are shown in Fig. S3(b).

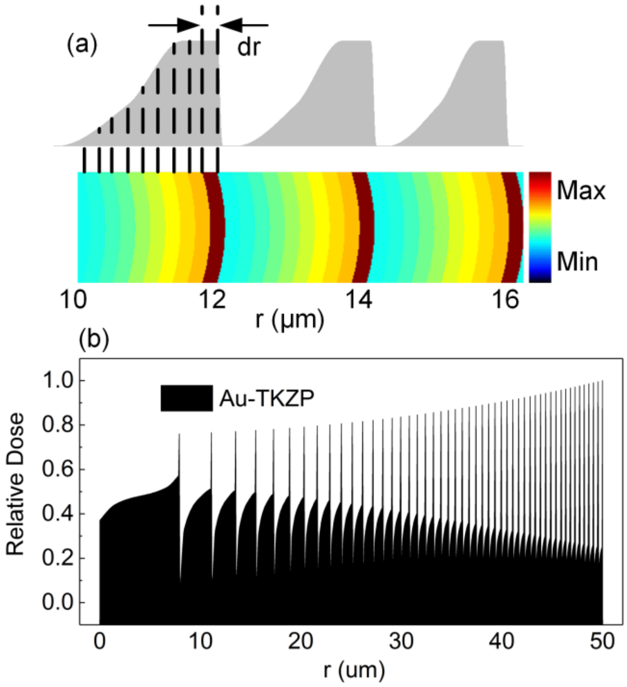

Figure S2 The optimized dose distributions for generating 3D profiles of the Trapezoid Kinoform lens. (a) According to the desired height (top), each zone was divided into layers and each of them was allocated with an exposure dose (middle) in order to form the desired 3D profiles. (b) the optimized dose distributions for Au-TKZP.

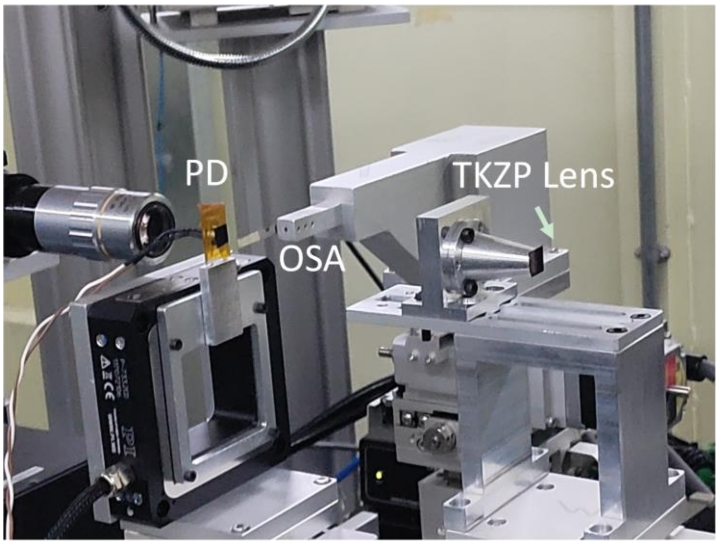

Figure S3 The optical-characterization setup used in this work.

Table S1 Design parameters for lenses demonstrated in this work. The diameter of beam stop is 50 μm for all FZPs in this work.

Lens	Au-FZP	Au-KZP	Au-TKZP
Material	Au	Au	Au
Energy (eV)	8000	8000	8000
Radius (μm)	50	50	50
Focal Length (mm)	161	161	161
Outmost zone width (nm)	500	500	500
Structure factor	0.5	0	0.2
Height (μm)	1.5	1.8	1.8
Exposure unit ring width (nm)	50	50	50
